# The first wave of the SARS-CoV-2 epidemic in Tuscany (Italy): A SI^2^R^2^D compartmental model with uncertainty evaluation

**DOI:** 10.1371/journal.pone.0250029

**Published:** 2021-04-21

**Authors:** Michela Baccini, Giulia Cereda, Cecilia Viscardi

**Affiliations:** Department of Statistics, Computer Science, Applications (DiSIA), University of Florence, Florence, Italy; Italian National Research Council (CNR), ITALY

## Abstract

With the aim of studying the spread of the SARS-CoV-2 infection in the Tuscany region of Italy during the first epidemic wave (February-June 2020), we define a compartmental model that accounts for both detected and undetected infections and assumes that only notified cases can die. We estimate the infection fatality rate, the case fatality rate, and the basic reproduction number, modeled as a time-varying function, by calibrating on the cumulative daily number of observed deaths and notified infected, after fixing to plausible values the other model parameters to assure identifiability. The confidence intervals are estimated by a parametric bootstrap procedure and a Global Sensitivity Analysis is performed to assess the sensitivity of the estimates to changes in the values of the fixed parameters. According to our results, the basic reproduction number drops from an initial value of 6.055 to 0 at the end of the national lockdown, then it grows again, but remaining under 1. At the beginning of the epidemic, the case and the infection fatality rates are estimated to be 13.1% and 2.3%, respectively. Among the parameters considered as fixed, the average time from infection to recovery for the not notified infected appears to be the most impacting one on the model estimates. The probability for an infected to be notified has a relevant impact on the infection fatality rate and on the shape of the epidemic curve. This stresses the need of collecting information on these parameters to better understand the phenomenon and get reliable predictions.

## 1 Introduction

The SARS-CoV-2 epidemic, the new coronavirus strain identified by the Chinese authorities in early January 2020, is characterized by strong contagiousness which translates into a fast growth of the number of infected individuals. The serious complications due to COVID-19, the respiratory disease caused by the new coronavirus, may require hospitalization in intensive care units and lead to death, especially among the elderly and those affected by multiple co-morbidities.

Italy was the first European country to be hit by the epidemic. Since the first case of infection was reported in the Lodi/Codogno area on February 21st, the situation in Italy has evolved rapidly, with the largest number of confirmed cases and deaths in the Northern regions, where the pressure on the health system has been very strong. Following the example of China, Italy, as well as most European countries, has implemented progressive measures of social distancing starting from small areas limited to the first cases of infection, up to a complete lock-down extended to the whole nation. From March 9th to May 4th, the restrictions have been progressively intensified and citizens were prohibited from leaving home except in cases of proven need or urgency.

The process of spreading of an infectious disease is very complex and depends on countless factors. However, under some assumptions, its dynamics can be simplified and reproduced through mathematical models. A well-known class of models is the one of the compartmental models, based on the assumptions that at any time point during the epidemic the population is divided into compartments, i.e. groups of individuals who are in the same state, and that the transitions from one state to another follow simple probabilistic rules [1]. The most famous example of compartmental model is the SIR model that divides the population into Susceptible (individuals that are exposed to the risk of infection), Infected, and Resolved (individuals that have either recovered or died) [2]. Through compartmental models, one can obtain information about key quantities regulating the epidemic spreading and make short or long-term forecasts, allowing comparative assessment of alternative policies to be adopted to face the epidemic [2, 3].

Since the beginning of the emergency, several approaches based on compartmental models have been proposed to study the SARS-CoV-2 epidemic, also in the Italian context [4–7]. The interested reader can find an accurate description of the literature in [8]. Some of the proposed compartmental models explicitly account for the presence of undetected asymptomatic individuals, thus for the fact that part of the epidemic is submerged [9–15].

The present paper is placed in this strand of literature, with the aim of studying the spread of the SARS-CoV-2 infection in Tuscany, a region in the center of Italy (3’737’000 inhabitants), during the first wave of the epidemic. With this purpose, we use a compartmental model which generalizes the classical Susceptible-Infected-Recovered-Deceased (SIRD) model [16, 17], defining distinct compartments for notified and not notified infected and for notified and not notified recovered, hence the acronym SI^2^R^2^D. Calibrating the model on two targets, the daily cumulative counts of deaths with COVID-19 and the daily cumulative counts of notified infections, we investigate the change of the basic reproduction number from the very early stage of the outbreak—before the introduction of the restriction policies—to the beginning of the summer season on June 20th, 2020. Additionally, we obtain estimates of both case fatality rate and infection fatality rate [18], after combining literature and local data to derive a value of the probability for an infected person to be notified. Estimates of the size of the compartments over time are produced as well.

A crucial point in compartmental models concerns parameter identifiability [19]. Especially in complex models including many compartments, the number of parameters to be estimated can be high and the data could be not sufficient to guarantee practical identifiability [20]. Additionally, different combinations of parameters could lead exactly to the same predictions, a situation which is usually called “theoretical non-identifiability”. In our model, to assure practical and theoretical identifiability, we leave unknown only a subset of the parameters, fixing the others to values obtained from the literature. This poses the problem of evaluating the sensitivity of the results to variations of the values used for the fixed parameters. So, we use the Global Sensitivity Analysis (GSA) to assess the relevance of the fixed parameters in determining the calibration results [21]. The GSA, which is not widely used in epidemiology and is rarely employed for compartmental models [22, 23], appears as one of the five recommendations in the manifesto “Five ways to ensure that models serve society: a manifesto” proposed by Saltelli and colleagues in a recent commentary which offers a critical view of modeling in time of pandemic [24].

## 2 Data

We use the data made available on a daily basis from February 24th, 2020 by Protezione Civile [25]. This database collects the daily numbers of notified positive, hospitalized, recovered, and deceased subjects by region. For our analysis, we focus on the cumulative daily number of COVID-19 deaths and on the cumulative daily number of notified infections in Tuscany up to June 20th, 2020.

## 3 Materials and methods

### 3.1 The SI^2^R^2^D model

**Fig 1:**
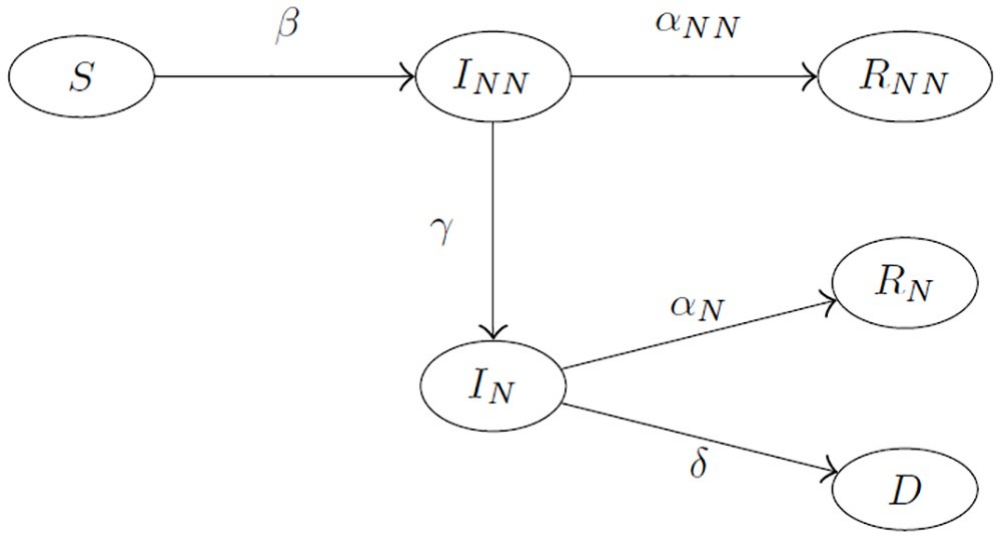
Admitted transitions between compartments of the SI^2^R^2^D model with the corresponding transition parameters.

Our model assumes that at any given time the population is divided into six compartments—Susceptible (*S*), Not Notified Infected (*I*_*NN*_), Notified Infected (*I*_*N*_), Not Notified Recovered (*R*_*NN*_) (recovered without being previously detected as infected), Notified Recovered (*R*_*N*_) (recovered after being previously detected as infected), Deceased (*D*)—and that the individuals can move between compartments according to the admissible transitions reported in Fig 1. We indicate with *S*(*t*), *I*_*NN*_(*t*), *I*_*N*_(*t*), *R*_*NN*_(*t*), *R*_*N*_(*t*), and *D*(*t*) the number of subjects belonging to the six compartments at time *t* = 0, 1, 2, …, measured discretely on a daily basis. Notice that individuals in *I*_*NN*_ can be either notified as infected or remain undetected by the authorities.

The model, that we call SI^2^R^2^D due to the presence of two compartments for the infected and two compartments for the recovered individuals, relies on the following assumptions:

1. The epidemic starts with one infected individual and the rest of the population susceptible, i.e. *I*(0) = 1 and *S*(0) = *N* − 1, where *N* is the regional population size.
2. The population is closed (we ignore demographic turnover, immigrations and emigrations): *S*(*t*) + *I*_*NN*_(*t*) + *I*_*N*_(*t*) + *R*_*NN*_(*t*) + *R*_*N*_(*t*) + *D*(*t*) = *N*, ∀*t*.
3. The population is homogeneously mixed and people make contact at random.
4. The transition parameters are constant across individuals who are present at the same time in the same compartment.
5. An individual entering the compartment *I*_*NN*_ on day *t* is supposed to be able to infect individuals belonging to *S* starting from time *t* + 1 until the transition to *R*_*NN*_ or *I*_*N*_. We assume in fact that individuals entering the compartment *I*_*N*_ are no more able to spread the contagion since they are detected and placed in isolation.
6. Recovered individuals cannot go back to being susceptible within our study period (the re-infection rate is equal to zero).
7. Only infected who are notified can die with COVID-19 (only asymptomatic or mild infections are eventually left undetected).

According to these assumptions, the dynamics underlying the model are described by the following equations:

$$\begin{array}{c} \left\{ \begin{array}{c} S\left(t\right)=S\left(t-1\right)-\beta \frac{S(t-1)}{S(0)}I_{NN}\left(t-1\right)\\ I_{NN}\left(t\right)=I_{NN}\left(t-1\right)+\beta \frac{S(t-1)}{S(0)}I_{NN}\left(t-1\right)-\gamma I_{NN}\left(t-1\right)-\alpha_{NN}I_{NN}\left(t-1\right)\\ I_{N}\left(t\right)=I_{N}\left(t-1\right)+\gamma I_{NN}\left(t-1\right)-\alpha_{N}I_{N}\left(t-1\right)-\delta I_{N}\left(t-1\right)\\ R_{NN}\left(t\right)=R_{NN}\left(t-1\right)+\alpha_{NN}I_{NN}\left(t-1\right)\\ R_{N}\left(t\right)=R_{N}\left(t-1\right)+\alpha_{N}I_{N}\left(t-1\right)\\ D\left(t\right)=D\left(t-1\right)+\delta I_{N}\left(t-1\right) \end{array}\right. \end{array}$$ (1)

where *t* = 0, 1, 2, …, and *β*, *γ*, *α*_*N*_, *α*_*NN*_, *δ* are the parameters which regulate the transitions, in the form of daily rates.

It is worth pointing out that in our analysis we use a model parametrization, detailed in S1 Appendix, which expresses the transition rates *γ*, *α*_*NN*_, *α*_*N*_, and *δ* as functions of:

- *π*, the probability of being notified for an infected subject, which is related to *γ* and *α*_*NN*_;
- *h*, the value of the case fatality rate, i.e. the probability of dying for a notified infected subject, which is related to *δ* and *α*_*N*_;
- the average transition times between compartments, $T_{R_{NN}}$, $T_{R_{N}}$, $T_{I_{N}}$, and *T*_*D*_, described in Table 1.

**Table 1:** Values of the transition times used in the SI^2^R^2^D model^a^.

Time	From	To	value
*τ*_1_	infection	symptoms	5 days [31–33]
*τ*_2_	symptoms	test	4 days [27]
*τ*_3_	symptoms	death	11 days [34]
*τ*_4_	symptoms	recovery	28 days [32]
$T_{I_{N}}=\tau_{1}+\tau_{2}$	infection	test	9 days
*T*_*D*_ = *τ*_3_ − *τ*_2_	test	death	7 days
$T_{R_{N}}=\tau_{4}-\tau_{2}$	test	recovery	24 days
$T_{R_{NN}}$	infection	recovery (for undetected)	14 days

^a^ The upper part of the table shows the literature values used to derive the transition times reported in the lower part of the table.

We introduce these parameters since they are meaningful quantities to estimate and make it simpler to include in the estimation procedure external information from literature and local data, which may enhance model identifiability (Section 3.2).

Regarding the transition rate *β*, it can be expressed as a function of *α*_*NN*_, *γ*, and *R*_0_, the so-called basic reproduction number, via the following equation [16]:

$$\begin{array}{c} \beta =R_{0}\left(\alpha_{NN}+\gamma \right). \end{array}$$ (2)

*R*_0_ represents the number of secondary infections that are expected to originate from the only positive person at the beginning of the epidemic. In our analysis we allow *R*_0_ to vary over time [26], inducing a time-varying *β*(*t*) via Eq (2). In particular, we assume that *R*_0_(*t*) is a piece-wise constant function with steps at times $t_{0}^{⋆}$, $t_{1}^{⋆}$, $t_{2}^{⋆}$, $t_{3}^{⋆}$:

$$\begin{array}{c} R_{0}\left(t\right)=r_{0}1\left\{t\in \left[0,t_{0}^{⋆}\right)\right\}+\sum_{j=1}^{3}r_{j}1\left\{t\in \left[t_{j-1}^{⋆},t_{j}^{⋆}\right)\right\}+r_{4}1\left\{t\in \left[t_{3}^{⋆},t_{e}\right]\right\}, \end{array}$$ (3)

where *t*_*e*_ is the number of days from the beginning of epidemic (*t* = 0) to June 20th. We a priori fix $t_{0}^{⋆}$, $t_{1}^{⋆}$, $t_{2}^{⋆}$, $t_{3}^{⋆}$ at the following dates: March 16th (the Monday after the start of the lockdown), April 6th, April 27th, and May 18th.

Finally, the model allows the case fatality rate (CFR) to decrease, starting from time $t_{2}^{*}$ (April 27th):

$$\begin{array}{c} \text{CFR}\left(t\right)=h1\left\{t\in \left[0,t_{2}^{⋆}\right)\right\}+\frac{h}{k}1\left\{t\in \left[t_{2}^{⋆},t_{e}\right]\right\}, \end{array}$$ (4)

where *k* ≥ 1. This implies that the infection fatality rate (IFR), which represents the risk of death among the infected individuals, decreases too, as it is obtained as the product of CFR(*t*) and *π*:

$$\begin{array}{c} \text{IFR}\left(t\right)=\pi \text{CFR}\left(t\right)=p1\left\{t\in \left[0,t_{2}^{⋆}\right)\right\}+\frac{p}{k}1\left\{t\in \left[t_{2}^{⋆},t_{e}\right]\right\}, \end{array}$$ (5)

where *p* = *πh*.

### 3.2 Model identifiability and calibration

In order to avoid identifiability problems [19], in our analysis we consider several parameters of the SI^2^R^2^D model as fixed. In particular, we fix *π* to a value obtained by combining literature estimates and local data (see Section 3.3), the transition times $T_{I_{N}}$, $T_{R_{NN}}$, $T_{R_{N}}$, *T*_*D*_ to the values reported in Table 1. While $T_{I_{N}}$, $T_{R_{N}}$, and *T*_*D*_ are derived from the literature after assuming an average time of 4 days from symptoms to infection detection in the notified cases (value based on Italian data [27]), $T_{R_{NN}}$ is fixed to 14 days—this is partly consistent with evidence indicating that the lowest viral load is reached 15 days after the infection onset [28]. Finally, we set *k* = 1.5, assuming that the case fatality rate and the infection fatality rate decrease by a 1/3 after April 27th [29, 30]. On the contrary, the parameters **r** = (*r*_0_, *r*_1_, …, *r*_4_) and *h* are left unknown and estimated through calibration as well as the date of the first infection. The infection fatality rate is derived from *π* and the estimate of *h* according to Eq (5).

Let us indicate with ***θ*** = (**r**, *h*) the vector of the unknown parameters. Given the fixed parameters, different values of ***θ*** correspond to different evolutions of the size of the compartments over time. Calibration is defined as the search for the value of ***θ*** producing the model trajectory that best matches target empirical data. In this work, as in [35], empirical data used for calibration are the observed daily time series of cumulative deaths (*D*_obs_) and of cumulative notified infections (*C*_obs_), both considered from March 9th, the day of the first observed death in the region, to the end of the study period on June 20th. For every ***θ***, we can simulate the corresponding time series *D*(*t*) and *C*(*t*)—the dependence of *D*(*t*) and *C*(*t*) on ***θ*** is omitted to ease notation—via the transition equations of the SI^2^R^2^D model. Notice that also the number of days from the first simulated infection to the first simulated death depends on ***θ***. Let D*, C* be the vectors obtained removing initial elements from *D* and *C* in order for their first element to correspond to the day of the first simulated death.

We minimize over ***θ*** the following weighted average of normalized root mean squared errors:

$$\begin{array}{c} Q=(w_{1}\frac{\sqrt{\sum_{t=1}^{T}{\left(D^{*}\left(t\right)-D_{\text{obs}}\left(t\right)\right)}^{2}}}{\sum_{t=1}^{T}D_{\text{obs}}\left(t\right)}+w_{2}\frac{\sqrt{\sum_{t=1}^{T}{\left(C^{*}\left(t\right)-C_{\text{obs}}\left(t\right)\right)}^{2}}}{\sum_{t=1}^{T}C_{\text{obs}}\left(t\right)}), \end{array}$$ (6)

where *T* is the number of days from March 9th to June 20th (104 days). Comparing D*(*t*) with *D*_obs_(*t*) and C*(*t*) with *C*_obs_(*t*), we constrain each simulated model to produce the first death on the same day when the first death is actually observed. This condition allows us to indirectly derive for each ***θ*** the date of the beginning of the epidemic.

In order to express a greater belief in the deaths counts, rather than in the observed notified infections, which are more prone to registration errors, false positives (albeit probably negligible) or delays, we a priori set *w*_1_ = 0.6 and *w*_2_ = 0.4 [11].

Minimization of Eq (6) is performed using the Hooke and Jeeves pattern search [36] through the function *hjn* in the R package optimR [37]. It is well-known that optimization methods in the context of compartmental models often depend on the chosen initial values of the algorithm [20, 38]. To avoid the problem of getting stuck in local minima we select the starting point through a preliminary minimization of *Q* on a multidimensional grid [19, 38].

The confidence intervals of the model parameters and of the size of the compartments are obtained through parametric bootstrap [39]. More precisely, we computed the percentile intervals according to the procedure proposed specifically for compartmental models by Chowell et al. [19]. We assume that the increments of the time series are distributed according to Negative Binomial distributions—a standard choice to model increments in mechanistic compartmental models [19, 40–44]—with parameters estimated by minimizing Eq (6) [40]. Then, we sample *n* = 1000 series of deaths and notified infections from these Negative Binomial distributions. Repeating the calibration procedure on each of the 1000 sampled pairs of series, we thus obtain 1000 bootstrap estimates of the model parameters ***θ*** and bootstrap 90% confidence intervals for the quantities of interest are calculated as the 5th and 95th percentiles of the distributions of these bootstrap replications [39]. Details about the procedure can be found in S1 Appendix, and its limitations are discussed in Section 5.2.

### 3.3 Estimation of *π*

Assuming that all infected subjects who undergo to SARS-CoV-2 test are correctly classified, the probability *π* for an infected individual to be notified is equivalent to the probability for an infected individual to be tested and can thus be written as *P*(*test*∣*infected*). Let *s*_*s*_, *s*_*m*_, and *s*_0_ be the events “the subject has severe symptoms COVID-19-like”, “the subject has mild symptoms COVID-19-like” and “the subject has not symptoms COVID-19-like”, respectively. The set {*s*_*s*_, *s*_*m*_, *s*_0_} is a partition of the event space *Ω*. The probability for a subject with ongoing infection to be tested can be decomposed as follows:

$$\begin{array}{cc} \pi & =P(notified|infected)=P(test|infected)=\\ & =P\left(test,s_{s}|infected\right)+P\left(test,s_{m}|infected\right)+P\left(test,s_{0}|infected\right)=\\ & =P\left(test|s_{s},infected\right)P\left(s_{s}|infected\right)+P\left(test|s_{m},infected\right)P\left(s_{m}|infected\right)+\\ & +P\left(test|s_{0},infected\right)P\left(s_{0}|infected\right)=\\ & =P\left(test|s_{s}\right)P\left(s_{s}|infected\right)+P\left(test|s_{m}\right)P\left(s_{m}|infected\right)\\ & +P\left(test|s_{0}\right)P\left(s_{0}|infected\right), \end{array}$$ (7)

where the last equality derives from the assumption that being tested is conditionally independent of the actual infection status given symptoms.

Relying on Eq (7) and using a Monte Carlo (MC) approach, we get an estimate of *π* and evaluate the uncertainty around it, combining information from the literature and local data (see Table 1). In particular, we consider as an estimate of *P*(*s*_0_ ∣ *infected*) the proportion of asymptomatic patients among the infected subjects (0.425%) observed by Lavezzo and colleagues [45] in the study aimed to detect, through molecular tests, the presence of SARS-Cov-2 infection in the small town of Vo’ (Italy). Due to the large percentage of participants (up to 85% of the residents), the descriptive data on the COVID-19 symptoms among the infected from this survey should not be affected by relevant selection bias related to the testing strategies adopted for detecting infections. Additionally, based on the assumption that all and only severe patients are hospitalized, we use the proportion of hospitalized subjects observed in the same study (0.16%) as an estimate of *P*(*s*_*s*_ ∣ *infected*). The estimate of the probability *P*(*s*_*m*_ ∣ *infected*) is obtained as one’s complement of the first two and results similar to that reported by a recent review [46].

We obtain estimates of *P*(*test* ∣ *s*_*m*_) and *P*(*test* ∣ *s*_0_) from the “IO CONTO” study, a survey conducted on students and staff of the University of Florence (Tuscany, Italy) in the months of April-May 2020 [47]. Due to the characteristics of the sample, we use this survey only to estimate the probability of being tested for subjects without symptoms or with mild symptoms (symptoms that did not require hospitalization). We consider as affected by COVID-19-like symptoms the respondents who declare to have experienced fever and/or cough and/or at least two of the following symptoms: respiratory problem, headache, diarrhea, vomit, asthenia, muscle pain, and loss of taste or smell [45]. According to this definition, we find an estimated value for *P*(*test* ∣ *s*_*m*_) equal to 0.017 (22 tested subjects over 1294) and an estimated value for *P*(*test* ∣ *s*_0_) equal to 0.021 (29 tested subjects over 1410). Finally, we assume that all people with severe COVID-19-like symptoms requiring hospitalization have been tested for SARS-CoV-2, i.e. *P*(*test* ∣ *s*_*s*_) = 1.

Given this information, we define two independent Beta distributions on *P*(*test* ∣ *s*_*m*_) and *P*(*test* ∣ *s*_0_) and a Dirichlet distribution on (*P*(*s*_0_ ∣ *infected*), *P*(*s*_*m*_ ∣ *infected*), *P*(*s*_*s*_∣*infected*)), with parameters estimated through the method of moments (Table 2). Then, we repeatedly sample from these distributions, calculating at each iteration, for a total of 10’000, a value of *π* according to Eq (7). In this way, we obtain an MC sample from the distribution of *π*. We use the average of this distribution as a fixed parameter in the calibration of the SI^2^R^2^D model; we use the entire distribution in the GSA.

**Table 2:** Estimated proportion and 5th and 95th percentiles of the Dirichlet/Beta distributions with parameters obtained via the method of moments.

	Ref.	Estimate	5th percentile	95th percentile
*P*(*s*_0_ ∣ *infected*)	Lavezzo et al. [45]	0.425	0.394	0.456
*P*(*s*_*s*_ ∣ *infected*)		0.16	0.138	0.184
. *P*(*s*_*m*_ ∣ *infected*)		0.415	0.384	0.446
*P*(*test* ∣ *s*_*m*_)	IO CONTO study	0.017	0.011	0.023
*P*(*test* ∣ *s*_0_)		0.021	0.015	0.027

We highlight that the value of *π* is assumed to be constant over time under the assumptions that the COVID-19 testing policies always rely on the same symptom-based criteria and that the distribution of the symptoms does not change over time during the study period (see also Russo et al. for a similar assumption [15]).

### 3.4 Global sensitivity analysis

In order to quantify the relevance of each parameter assumed as fixed in determining the calibration results, we perform a global sensitivity analysis (GSA) [21]. Given *K*_*X*_ mutually independent inputs $\left(X_{1},X_{2},...,X_{K_{X}}\right)$ and a model which, given the inputs, returns *K*_*Y*_ outputs $\left(Y_{1},Y_{2},...,Y_{K_{Y}}\right)$, the GSA explores how the outputs vary as the inputs change, with the aim of determining the most contributing input variables to the output behavior (factor prioritization), finding non-influential inputs (model simplification), and investigating interaction effects between inputs. This is done relying on Sobol’s decomposition of the variance of each output in the sum of variances of increasing order [48] (see S1 Appendix for further details).

The variance decomposition allows computing several indexes as described in S1 Appendix, including the total effect index for each input *X*_*i*_ and output *Y* (from here on, we suppress the subscript *i* of the output variable for sake of simplicity). This index represents the proportion of the total variance of *Y* which is due to the main effect of the input *X*_*i*_ and all its interactions with the other inputs. Denoted as $S_{i}^{\text{tot}}$, it is defined as

$$\begin{array}{c} S_{i}^{\text{tot}}=\frac{E(\text{Var}\left(Y|X_{∼i}\right))}{\text{Var}(Y)} \end{array}$$ (8)

where **X**_**∼i**_ denotes the vector $\left(X_{1},X_{2},...X_{i-1},X_{i+1},...X_{K_{X}}\right)$. According to the notation adopted in Saltelli [21], **X**_**∼i**_ is also the argument on which the outer operator E is applied to.

In the case of a multivariate output (*K*_*Y*_ > 1), an aggregated index can be obtained for each input as a weighted average of the single-output indexes, with weights proportional to outputs variances [49, 50].

In our application, we consider as inputs the fixed parameters of the SI^2^R^2^D model ($T_{R_{NN}}$, *T*_*D*_, $T_{R_{N}}$, $T_{I_{N}}$, *π*, and *k*) and, as outputs, the parameters estimated by calibration, as well as derived quantities, such as IFR, date of the first infection, and date of infection peak. The model is the calibration algorithm, given the observed data.

**Fig 2:**
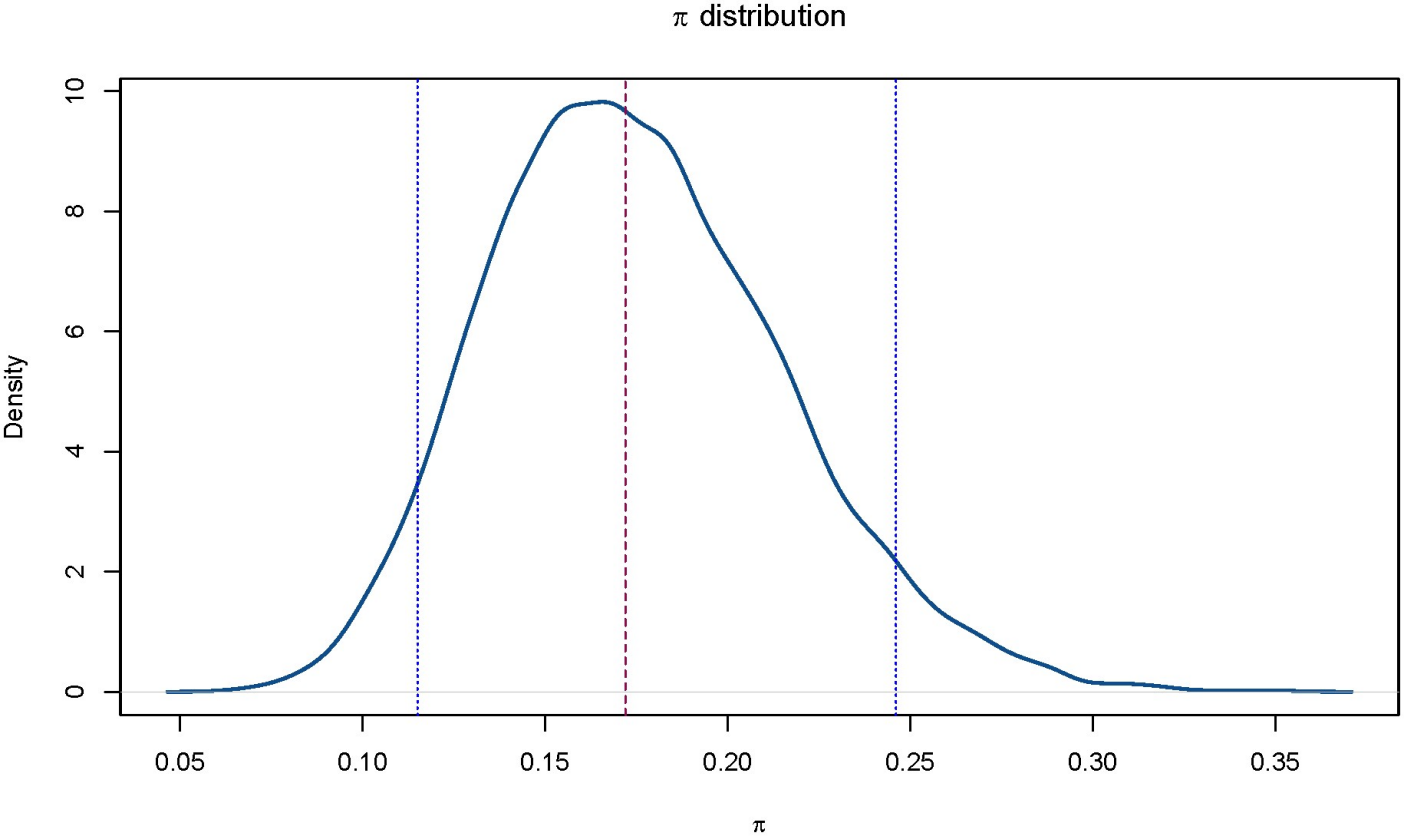
Monte Carlo approximation of the distribution of the probability *π* with median (dashed red line), 5th and 95th percentiles (dashed blue lines).

Sobol’s indexes are obtained through an MC approximation. We calculate for each input the total effect indexes on each output and the aggregated indexes on **r**, relying on the results of 8’000 calibrations. Each calibration is performed under a different combination of inputs, obtained by sampling from the empirical distribution of *π* (Section 3.3, Fig 2), from a continuous uniform distribution U[1,2] for *k* and from the following discrete uniform distributions for the transition times: $T_{R_{NN}}∼U\left\{7,21\right\}$, $T_{D}∼U\left\{3,14\right\}$, $T_{R_{N}}∼U\left\{14,40\right\}$, $T_{I_{N}}∼U\left\{3,14\right\}$.

The GSA is conducted by using the *soboljansen* and the *sobolMultOut* functions of the R package sensitivity [51].

## 4 Results

The average of the MC distribution of *π*, obtained according to the procedure described in Section 3.3, is 0.175 and the 5th and 95th percentiles are 0.115 and 0.246, respectively (see also Fig 2).

**Fig 3:**
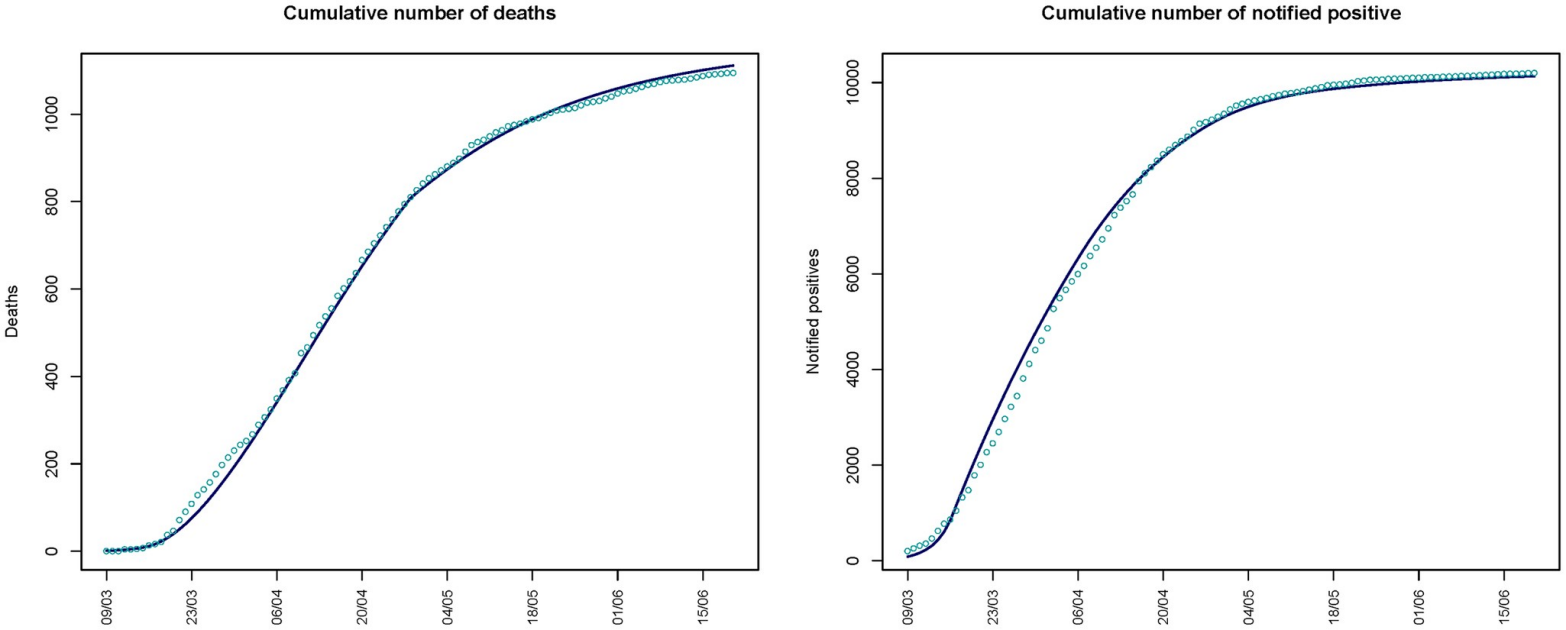
Cumulative number of deaths and number of notified infected circulating in the region estimated from the SI^2^R^2^D model (continuous lines) and observed (points).

As shown in Fig 3, the fit of the SI^2^R^2^D model is quite good, with the expected cumulative time series *C* and *D* close to the observed ones. Table 3 reports the estimated values of the unknown model parameters. From the beginning of the epidemic, estimated to be February 14th, to April 27th, the estimated case fatality rate, *h*, and the estimated infection fatality rate, *p*, are 0.131 (90% CI 0.121, 0.141) and 0.023 (90% CI 0.021, 0.025), respectively. Then, these rates decrease to 0.087 and 0.015, according to the fact that we fix *k* = 1.5.

**Table 3:** Estimates of the unknown parameters of the SI^2^R^2^D model with 90% confidence intervals.

	Time period	Estimate	IC90%	
**r**_**0**_	Before March 16th	6.055	6.006	6.099
**r**_**1**_	March 16th—April 5th	0.722	0.642	0.819
**r**_**2**_	April 6th—April 26th	0.393	0.318	0.46
**r**_**3**_	April 27th—May 17th	0	0	0.164
**r**_**4**_	May 18th—June 20th	0.493	0	0.669
**h**		0.131	0.121	0.141

**Fig 4:**
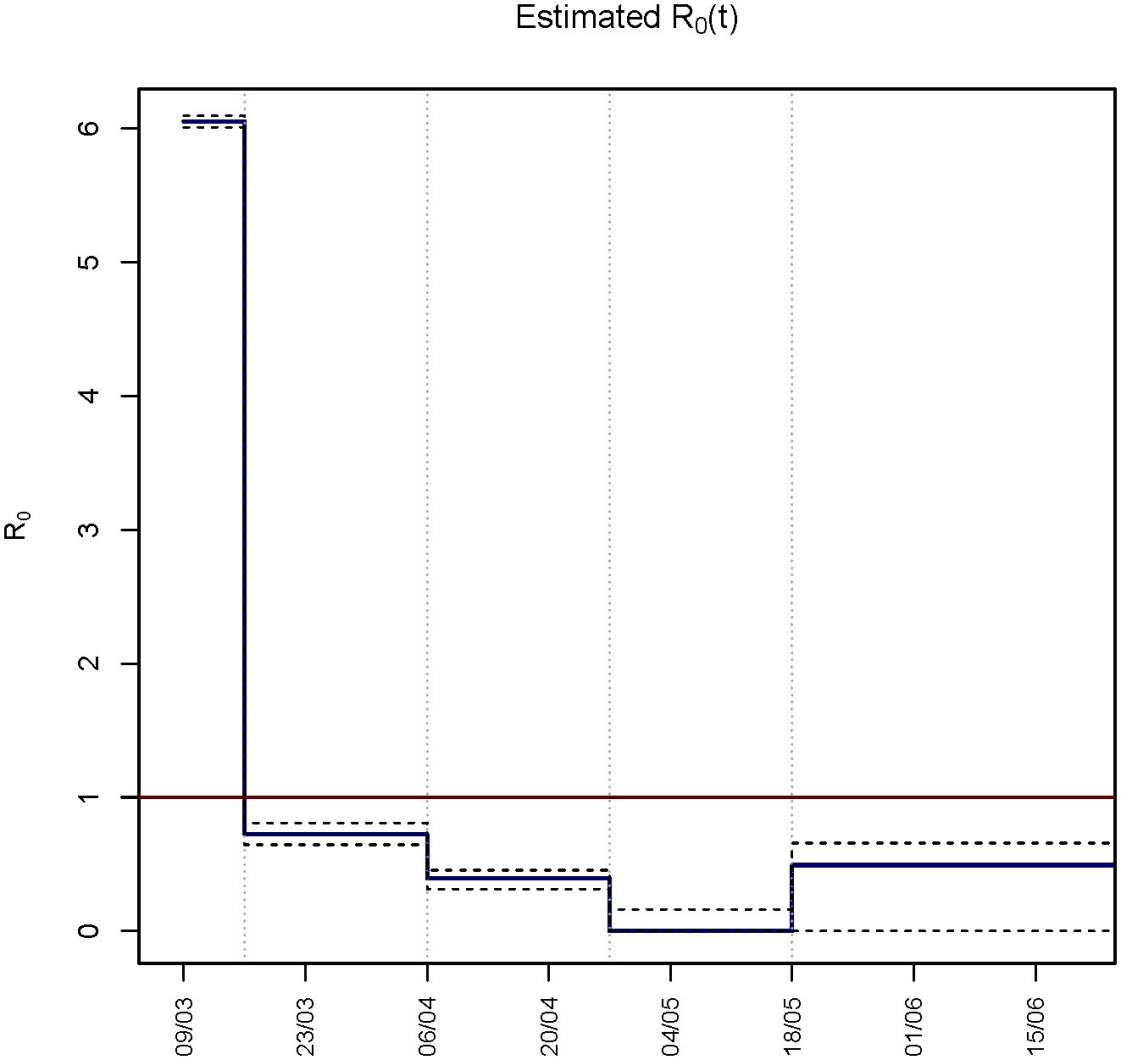
Estimated *R*_0_(*t*) with 90% confidence intervals.

The basic reproduction number drops from an average value of 6.055, estimated for the period before March 16th, to 0.722 (90% CI 0.642, 0.819) from March 16th to April 5th. After this date it continues to fall reaching 0.393 (90% CI 0.318, 0.46) in the period April 6th- April 26th, and then 0 (90% CI 0, 0.164) in the period April 27th-May 17th. Thereafter, it seems to grow again with an estimated value of 0.493 (90% CI 0, 0.699) (Table 3 and Fig 4). The confidence intervals around the last two steps of *R*_0_(*t*) are quite large, suggesting a certain degree of uncertainty which is likely due to the scarce information on infection transmission when the number of new cases is low.

**Fig 5:**
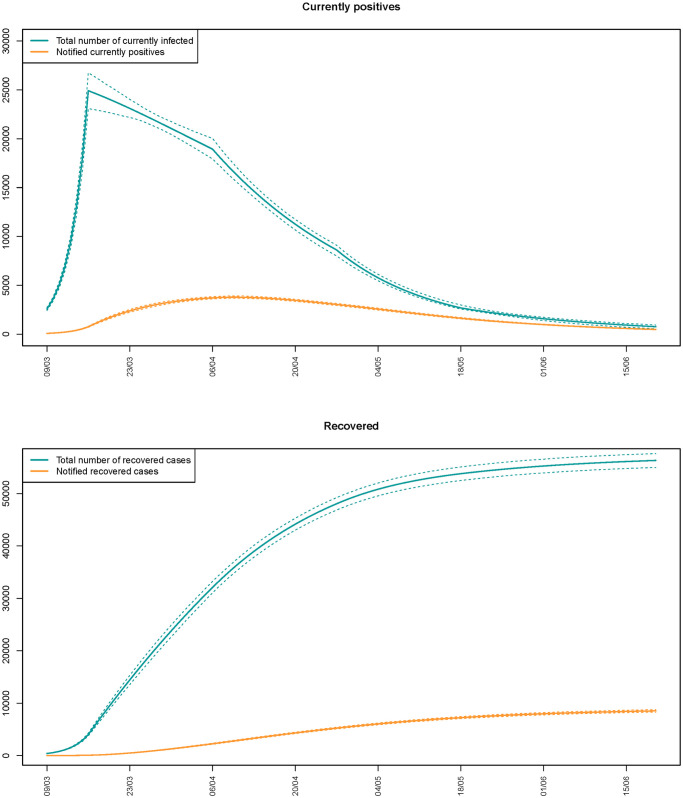
Evolution in time of the total daily number of currently infected and recovered individuals, part of whom are notified (orange line).

In the upper panel of Fig 5 we report the change over time of the total daily number of the currently infected individuals, part of whom are notified (orange line in the graph). The peak of the epidemic (the day with the largest number of circulating infections) is estimated on March 16th, with a number of circulating infections in the region equal to 24’918 (90% CI 23’086, 26’754). This peak does not correspond to the peak for the notified infections, which is estimated to happen 25 days later, on April 10th. In Fig 5 (lower panel), the cumulative number of recovered individuals (total and notified) is shown as well.

Let us call back that the inputs of the GSA are the fixed parameters of the SI^2^R^2^D model, while the outputs are the unknown ones. Furthermore, we compute the total variance indexes for three derived quantities: date of the first infection, date of the infection peak, initial infection fatality rate *p*. Table 4 reports the total variance indexes by single output. The transition time $T_{R_{NN}}$ turns out to be the most impacting factor on the single components of **r**, followed by $T_{R_{N}}$ and $T_{I_{N}}$ which are particularly influential on *r*_1_ and *r*_3_. With the exception of *r*_0_, the values of the basic reproduction numbers are slightly influenced by *k* and *π* as well. In order to evaluate the impact of the inputs on the basic reproduction number as a whole, we calculate the aggregate total variance indexes for the vector **r** (last column in Table 4). These indexes indicate that $T_{R_{NN}}$ is the most impacting input on *R*_0_(*t*) (aggregate index equal to 0.918). The relevance of $T_{R_{NN}}$ on the estimate of *R*_0_(*t*) is confirmed even when we exclude from the aggregate index calculation *r*_1_, which is the element of **r** with the largest variance, thus the largest weight in the aggregate index calculation (aggregate index equal to 0.754). The parameter *h* is mostly influenced by $T_{R_{N}}$ and to a lesser extent by *k*.

**Table 4:** Total variance indexes of each model input (by row) on the model outputs (by column); aggregated total variance indexes on r.

	h	r_0_	r_1_	r_2_	r_3_	r_4_	Aggregated for r	p	First infection	Peak
**k**	0.230	0.017	0.313	0.216	0.261	0.315	0.077	0.039	0.140	0.537
**π**	0.030	0.03	0.353	0.112	0.545	0.136	0.057	0.039	0.140	0.537
**T**_**D**_	0.016	0.008	0.138	0.045	0.181	0.047	0.018	0.002	0.062	0.314
$T_{R_{N}}$	0.788	0.030	0.715	0.172	1.034	0.238	0.081	0.128	0.417	1.014
$T_{R_{\text{NN}}}$	0.026	0.963	0.646	0.812	0.845	0.747	0.918	0.005	0.724	0.829
$T_{I_{N}}$	0.028	0.052	0.466	0.186	0.700	0.145	0.080	0.004	0.260	0.885

Values of the total index slightly exceeding 1 are due to MC approximation.approximation.

Looking at the derived quantities one can see that the date of the peak is particularly sensitive to variations of $T_{R_{N}}$, $T_{I_{N}}$, $T_{R_{NN}}$, and *π*. The date of the first infection is mostly influenced by $T_{R_{NN}}$ while the only parameter that seems to have a relevant impact on *p* is *π*.

Considering that the sum of the total variance indexes relative to the same output exceeds 1, we can conclude that there is a relevant interactions effect among the inputs (see S1 Appendix).

**Fig 6:**
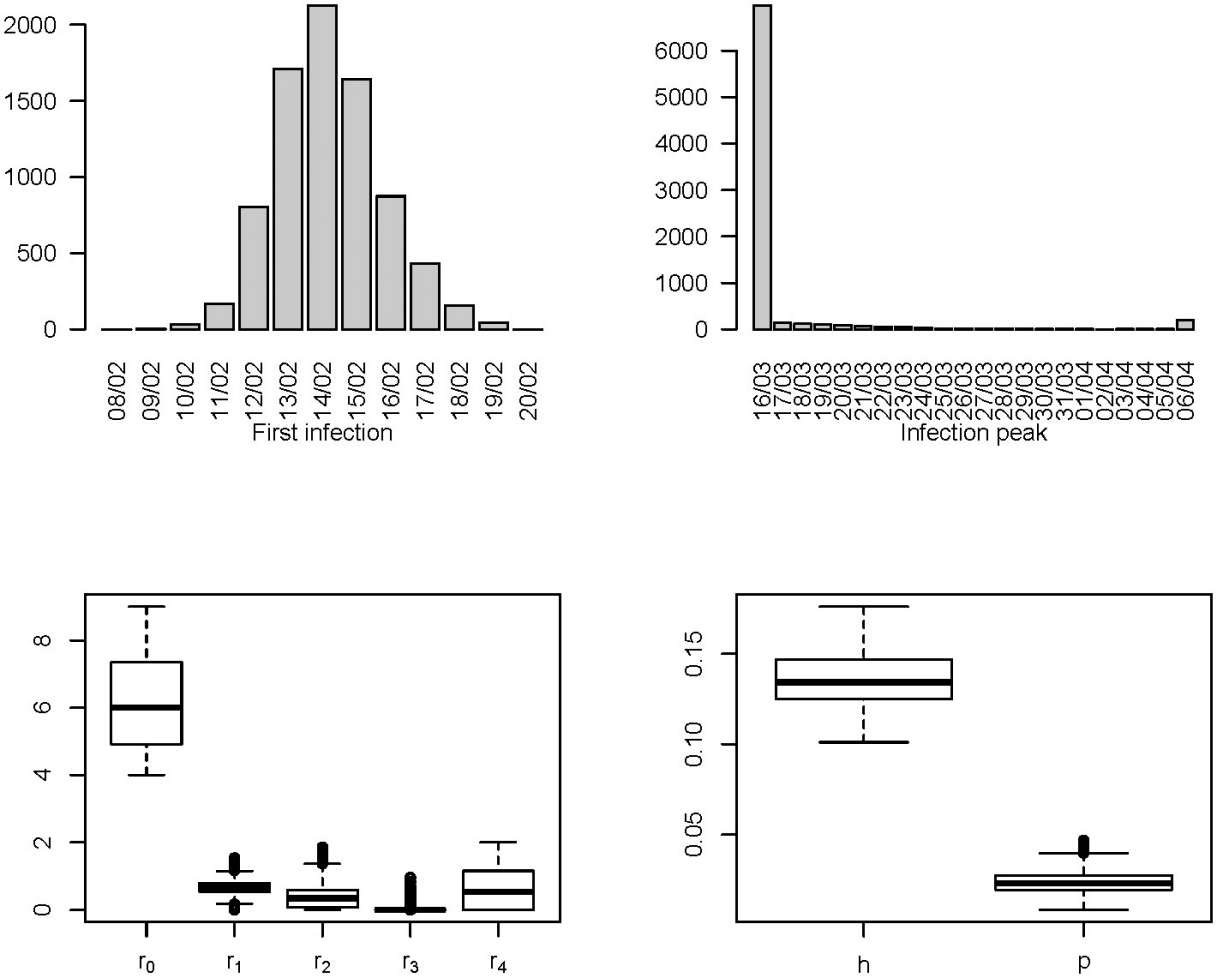
Distributions of the model outputs as the inputs vary.

In Table 5, we summarize in terms of mean, median, 5th and 95th percentiles the distributions of the outputs arising from the 8’000 simulations of the GSA. The same distributions are shown in the bar charts and box plots in Fig 6. They express the variability of the reported outputs, which propagates over them from the uncertainty around the parameters which are considered as fixed. This variability should be not confused with the sampling variability and the reported percentiles do not represent the extremes of confidence intervals.

**Table 5:** Mean, median, 5th, and 95th percentiles of the model outputs as the inputs vary.

	Mean	Median	5th percentile	95th percentile
**h**	0.136	0.134	0.115	0.159
**r**_**0**_	6.106	5.997	4.000	8.462
**r**_**1**_	0.652	0.6739	0.271	0.912
**r**_**2**_	0.382	0.346	0.000	1.017
**r**_**3**_	0.020	0.000	0.000	0.169
**r**_**4**_	0.636	0.528	0.000	1.792
**p**	0.024	0.023	0.015	0.035
**First infection**	February 14th	February 14th	February 12th	February 17th
**Infection peak**	March 17th	March 16th	March 16th	March 23rd

## 5 Discussion

The results indicate that in Tuscany the basic reproduction number changed over time during the study period, reaching its minimum around the end of the national lockdown on May 4th, then slightly coming back to higher values from the half of May 2020. This descriptive pattern, which seems confirmed even when considering the uncertainty related to the variations of the fixed parameters in the SI^2^R^2^D model (Fig 6 and Table 5), is indicative that the lockdown has likely had a very strong effect on virus transmission, even if, on the basis of our analysis, it is not possible to exclude that other factors, such as for example the increase in air temperatures, have had a role in bringing the level of contagions at its minimum immediately after the end of the lockdown [52, 53].

The fact that the estimate of *R*_0_(*t*) becomes exactly zero between April 27th and May 18th deserves a discussion also in the light of the model assumptions. Being the population close and $T_{R_{NN}}=14$, the resumption of the epidemic from May 18th—which implies the presence of infectious individuals at that date—may appear at first glance paradoxical after 21 days of a null *R*_0_(*t*). However, this result is perfectly justified if one considers that our model implicitly assumes exponential distributions on the transition times [2], thus a not negligible probability that the time spent by an individual in the compartment of origin is much longer than the average. We cannot exclude that assuming on $T_{R_{NN}}$ a different probability distribution, narrower than the exponential around the mean, for example an Erlang distribution with a large shape parameter [54], could lead to a basic reproduction number above zero over the whole study period.

The value of the basic reproduction number estimated for the time window from the beginning of the epidemic to one week after the beginning of the national lockdown expresses the initial capacity of the SARS-CoV-2 to spread (in terms of the average number of people whom every infected person transmits the virus to), which depends not only on the virus characteristics but also on factors related to the social, demographic and economic context. These factors may include population density, level of daily commuting and use of public transport, social closeness, family structure, and composition. For this reason, a strong heterogeneity of the index across regions or countries is expected. Having said this, we estimate an initial *R*_0_(*t*) equal to 6.055, which could range between 4 and 8.462, according to the GSA. These estimates are quite high, but consistent with part of the literature that reports values of *R*_0_ at the beginning of the epidemic even larger than 6, in particular in high contact density situations, as during the outbreak on the Diamond Princess cruise ship [55]. For example, the review performed by Liu and colleagues [56] reports *R*_0_ values in China ranging from 1.4 to 6.49, with the highest estimates obtained when compartmental models are used. Not least, in comparing our estimate of the basic reproduction number with those reported elsewhere, it is necessary to consider that our model accounts also for the undetected cases, leading to a number of secondary infections caused by a carrier possibly higher than in analyses that exclusively focus on the notified cases [57, 58].

Our model estimates an initial CFR equal to 13.1% which, according to the GSA, could range from 11.5% to 15.9%. Then, we assume that after April 27th the CFR decreases by one-third. Evidence of a decreasing CFR, although still debated, is supported by some studies. For example, Pachetti and colleagues [29] document a decrease in the CFR during the month of April 2020 in several European countries, including Italy. A decrease in CFR is in line with a possible improvement of patients care after the first months of the emergency—partly as a consequence of a decreased pressure on the health system –, but also with less explored scenarios: changes of the typical COVID-19 patients, reduced population vulnerability as related to modified environmental conditions (e.g. raising air temperatures, lower air pollution level due to the lockdown), reduced virus aggressiveness [30]. An interesting hypothesis has been recently suggested by Gandhi and Rutherford [59]: population-wide masking could increase the percentage of asymptomatic infections, thus induce a reduction in the CFR, especially in regions and countries where the use of masks is mandatory—as in the Tuscany region during the study period.

Changes in the CFR could be also due to variations in the testing policies: more extensive testing campaigns could detect milder and asymptomatic cases and could do it earlier, thus increasing the chance of recovery for the notified cases [29]. However, relying on the fact that the regional and national guidelines about testing did not substantially change during the study period, we have a priori discarded this hypothesis, assuming that both *π* and *T*_*T*_ do not vary over time [60, 61].

The CFR estimated in our analysis is in line with that reported for Italy by Sartor and colleagues [62] and in the range of those reported for other Italian regions, but higher than that observed in other countries [61, 63]. The CFR obviously depends on the number of detected cases that, in turn, depends on the adopted testing policies, but possible explanations of the observed differences are also the different age structure of the population (Italy has one of the oldest population in Europe and fatal COVID-19 outcomes are mostly among the elderly) and the different definition of the COVID-19 deaths (in Italy the COVID-19–related deaths are all those occurring in SARS-CoV-2 positive patients regardless the role of possible previous diseases) [61]. The hypothesis that higher CFRs can be related to increased population frailty induced by long-term exposure to high air pollution levels should not be ruled out as well (see for example [64]).

We estimate an initial IFR value equal to 2.3% (between 1.5% and 3.5% from the GSA) that, like the CFR, decreases by one-third since April 27th. This estimate should be interpreted accounting for the same issues already highlighted for the CFR, with the difference that it measures the mortality among all infected individuals regardless of their notification status. The IFR estimate is strongly affected by the value of *π*, which, regulating the proportion between notified and undetected infections, allows calculation of IFR from CFR.

The estimate of *π* is obtained by combining information from different sources and relying on assumptions that are consistent with the guidelines issued by the regional government on SARS-CoV-2 testing during the study period, in particular with the fact that these guidelines relied on symptom-based criteria. Furthermore, the resulting distribution of *π* is coherent with the results of the SARS-CoV-2 seroprevalence survey initiated by the Italian Ministry of Health and Istat, which reports a ratio between notified infected and actually infected subjects of 1 over 6 in Italy and slightly higher in Tuscany [65]. This indicates that data about symptoms and testing, relatively simple to collect, might be a low-cost alternative to seroprevalence surveys in order to get estimates of the proportion of notified infections over the total.

The cumulative number of new infections from the beginning of the epidemic to the end of the study period estimated by the SI^2^R^2^D model corresponds to 1.4% of the regional population, slightly higher than the value (1%) obtained for the Tuscany region from the seroprevalence survey [65].

Interestingly, calibration leads to epidemic dynamics where the estimated peak of circulating infections in the region is earlier than the estimated peak for the notified cases. This result, which cannot be corroborated by empirical data, is likely related to the fact that in our model time from infection onset to recovery is assumed to be shorter in case of non-notification than in case of notification, in agreement with the literature.

The relevance of using mathematical modeling in facing health emergencies has emerged in recent months together with the dangers related to the false sense of certainty deriving from the quantitative evaluation of complex systems such as those regulating the epidemic dynamics [24, 66]. Models can provide estimates of meaningful quantities, short and medium-long term forecasts and can be used to assess the impact of actual or hypothetical policies and interventions [4, 26, 67–69]. On the other hand, the risk of over-interpreting their results is high and mostly related to unsatisfactory evaluation and communication of model uncertainties. Uncertainty evaluation in the form of GSA is not routinely used in the context of epidemic dynamics modeling, but examples exist in which Sobol’s variance decomposition and variance indexes are employed in the phase of model definition, in order to reduce model complexity by detecting the parameters which have a negligible impact on specific predictions (e.g. the infected population size at the peak of the epidemic) [22, 23].

In this paper we use the GSA in a different way, to evaluate how the uncertainty around the parameters taken as fixed in the SI^2^R^2^D model propagates and affects the calibration estimates of the parameters that are left unknown. In some sense, this is not far from the GSA-GLUE approach, which is a version of GSA conditioned to observations, where a distance measure between observed and predicted data is estimated at each run of the GSA, then a classification of the model runs is done according to this distance measure [70]. It is worth noting that, being each run of the GSA conditioned to the observed data, we decompose, according to Sobol’s formula, the variance of the point estimates of the unknown parameters as the inputs change, which does not include sampling variability. In fact, the sampling variability is quantified a part via bootstrap.

The GSA indicates that, in order to reduce outcomes variability, additional evidence would be needed on the transition times, in particular on the time from infection to recovery for the infected that are not notified, the only ones able to spread the contagion. The recovery time for these individuals, who are likely asymptomatic or paucisymptomatic, is here assumed to coincide with the duration of their infectious status. While a reliable estimate of this duration would be crucial also from a practical point of view—for instance to define an appropriate quarantine length for asymptomatic individuals who came into contact with infected -, the evidence on it is still limited [71].

The probability *π* has only a moderate impact on the point estimates of the basic reproduction number and CFR, but, given its role in tuning the relative size of notified and not notified compartments, has a high impact on the IFR and on the epidemic curve.

### 5.1 External validity of the model

Applying the SI^2^R^2^D model proposed in this paper to different contexts requires a critical discussion of the main assumptions which it relies on [24].

First of all, performing analyses at a regional or sub-regional level, as in this paper, instead of at a national one is to be preferred if the parameters that regulate the epidemic dynamics are heterogeneous. On the other hand, the assumption of a closed population is all the more critical the smaller is the study area, unless there are conditions of reduced mobility that minimize the possibility of inter-regional contagion, as during the national lockdown and more in general during the study period (the national borders were kept closed until the half of June when they have been progressively re-opened). This aspect should be accounted for in defining the study area.

Second, our SI^2^R^2^D model includes two infected and two recovered compartments while only one deceased compartment is specified, because we assume that the undetected infections did not bring to major health problems, nor to death. This assumption, already used in [72] and reasonable for the Tuscany region, could be inappropriate if the model is adopted to investigate the epidemic dynamics in areas where, being the health service under stress, part of the COVID-19 mortality ended up being not notified, as happened in the Northern Italian regions during the first epidemic wave [73, 74]. Similarly, it could be needed to modify the transition equations in order to allow a certain level of contagion coming from the notified infected, if there is evidence of ineffective isolation of the individuals that are notified as infected (violation of assumption 5). This eventuality, which is excluded also by other authors who used similar compartmental models [15], seems to be remote for the Tuscany region during the study period, but it could be more plausible in other contexts, depending on the level of preparedness of the health care system, the availability of personal protective equipments for population and health care workers and of adequate and sufficient facilities to ensure patient isolation.

A third relevant assumption is that the re-infection rate is equal to 0, consistently with a long-term immunity theory, which however is still to be confirmed [75]. Being the size of the susceptible compartment extremely large at the beginning of the epidemic, the effect of a violation of this assumption is negligible for the purpose of the present study. However, this could be not the case at later stages of the epidemic or if the focus is on a longer study period.

Regarding the calibration procedure, additional targets could be considered. In our analysis, we calibrate on the observed cumulative time series of deaths and of notified infected, but in principle, the observed cumulative time series of the notified recovered could be considered as well, if of good quality. More in general, the weights used for the calibration targets should be defined according to the actual data reliability.

### 5.2 Study limitations

Our study has several limitations. Here, we highlighted the main ones. The first one concerns the estimation procedure. We estimate the parameters in a deterministic way, in the sense that the calibration estimates are obtained without making distributional assumptions on the data. Successively, a Negative Binomial distribution is used to generate bootstrap samples and to quantify sampling uncertainty, but this probability distribution does not enter in calibration. This kind of procedure is quite usual in compartment models literature, since modeling data through stochastic differential equations results in the definition of very complex likelihood functions [19, 20]. However, more sophisticated alternatives exist. Among these, calibration via Approximate Bayesian Computation has been recently proposed as a tool to make Bayesian inference on stochastic compartmental models, bypassing the definition of the likelihood and allowing to take into account also the uncertainty around parameters values [76].

The bootstrap procedure adopted for obtaining the confidence intervals has some limitations. In particular, still preserving the allowable parameter range [39, Ch 13], which is a useful property in our context, percentile intervals have lower coverage than intervals obtained through other procedures. Better coverage could be get by calculating iterated bootstrap confidence intervals or bias-corrected and accelerated bootstrap confidence intervals. However, both solutions appear too complex or computationally demanding in our setting [77], as well as performing simulations aimed at assessing the frequentist coverage of the intervals. A different procedure for confidence intervals calculation in compartmental models can be found in [78].

Our procedure fixes the date of the first infection by aligning the simulated time series of deaths with the observed one, so that the time of the first simulated death coincided with the time of the first observed death. This procedure is probably responsible for an underestimation of the sampling variability around the date of the onset of the epidemic in the region and around the initial value of the basic reproduction number.

The basic reproduction number and the CFR are assumed to vary over time according to piece-wise functions with steps at fixed calendar days. Flexible modeling, for example through regression splines, could provide more realistic results. Additionally, the percent of decrease of CFR, thus of IFR, is a priori fixed on the basis of empirical evidence, and not estimated from the epidemic data used for calibration.

Although we are confident that the reported total variance indexes correctly describe the actual impact of the model inputs on the outputs, a formal evaluation of the convergence of the indexes estimate could be useful [79]. As this evaluation would require an additional important computational effort, we reserve the right to address this issue in an *ad hoc* study.

Finally, assumption 3 of the SI^2^R^2^D model states that the average transition rates are the same for all individuals belonging to the same compartment at a certain time *t*. Similarly, it assumes that all individuals have the same chance to come into contact with each other, ruling out, for example, the possibility that contacts are more frequent within specific subgroups and in general that virus transmission follows pathways reflecting the complex structure of the population. Compartmental models that stratify by age could be the first step to relax this assumption [80].

### 5.3 Comparison with similar models

With the aim of taking into account the undetected infected, our model includes more compartments than a standard SIRD. In the same spirit of our work, other authors proposed compartmental models allowing for the presence of undetected asymptomatic individuals in the population [9–15]. Calafiore and colleagues [12] resort to a standard SIRD model and take into account the undetected infected individuals assuming that they represent a fixed fraction of the notified ones, as we do. However, they also assume that the rate of recovery and the rate of death are the same among detected and undetected infected individuals. Our SI^2^R^2^D model overcomes this strong assumption by introducing two further compartments. Quaranta and colleagues [11] define a compartmental model that, under the assumption that all and only the symptomatic cases are detected, is very similar to ours. Like us, they also consider a time-varying infection rate and calibrate the model on more than one target quantity. A key feature of our proposal, when compared with theirs, is the choice of a parametrization that allows for directly estimating meaningful quantities such as the basic reproductive number and the fatality rates.

Also, the very recent model by Russo et al. [15] has the same compartments as our SI^2^R^2^D model. However, as Quaranta et al. [11] and most of the other referred authors, it does not take into account sampling uncertainty and only gives point estimates of the unknown parameters. An approach incorporating sampling uncertainty has been proposed in Kucharski et al. [81]. Interestingly the same authors also emphasize the need to perform sensitivity analyses to evaluate the robustness of the model results. This is a crucial point in compartmental models. In most applications, key quantities on which solid evidence is not yet available, such as transition times between compartments and infection fatality rates, are actually assumed as fixed without performing any robustness or uncertainty evaluation [45]. In Sen et al. [13] a sensitivity analysis is performed through comparisons among simulated scenarios corresponding to different parameter values. In our paper, we formally address this issue by performing a GSA that provides a global evaluation of the robustness of our estimates when the fixed quantities change within plausible ranges of values.

To wrap up, the main contributions of our paper are: the formulation of a compartmental model which takes account for undetected infections; the use of a parametrization allowing for a direct estimate of epidemiological meaningful quantities such as reproduction number, IFR, CRF; the quantification of uncertainties. Specifically, we account for sampling variability by estimating percentile confidence intervals through a bootstrap procedure and we quantify the structural uncertainty related to having fixed the values of key parameters through the GSA.

## 6 Conclusions

In this paper, we use a compartmental model to estimate meaningful quantities such as basic reproduction number, CFR, and IFR during the first wave of the SARS-CoV-2 infection in an Italian region. Our results indicate that the virus transmission, very high at the beginning of the epidemic outbreak, strongly decreased immediately after the introduction of the national lockdown and that *R*_0_(*t*) remained below the threshold of 1 up to the beginning of the summer season. We find as well that in the first months of the epidemic wave the 2.3% of the infected died, corresponding to 13.1% of the notified cases. The observed data are consistent with a possible decrease of the COVID-19 fatality from the end of April.

The need to fix some parameters to assure identifiability and the need to check the sensitivity of the results to the values used for the fixed quantities in the model are two faces of the same coin. The GSA is useful to investigate how the results change with the fixed parameters and indicates which are the most impacting ones, suggesting investigation priorities. For example, the estimate of *R*_0_(*t*) seems to be strongly influenced by the average waiting time from infection to recovery for the not notified infected. This suggests that a better definition of the infection duration in asymptomatic cases could enhance the understanding of the transmission dynamics.

The proposed model relies on strong assumptions that could be inappropriate in other areas and at different stages of the epidemic. Therefore, the opportunity of modifying the model structure should be considered in future applications.

## Supporting information

S1 Appendix Additional details on parameter definition, parametric bootstrap, Sobol’s decomposition of the variance. (PDF)
