# Correction: The first wave of the SARS-CoV-2 epidemic in Tuscany (Italy): A SI^2^R^2^D compartmental model with uncertainty evaluation

**DOI:** 10.1371/journal.pone.0252908

**Published:** 2021-06-02

**Authors:** The PLOS ONE Staff

There are errors in Table 4 which were introduced in the typesetting process. The values found in the last three columns of row **k** (P, First infection, and Peak) were duplicated in row *π*. The publisher apologizes for the error. Please see the correct version of Table 4 here.

**Table 4:** Total variance indexes of each model input (by row) on the model outputs (by column); aggregated total variance indexes on r.

	h	r_0_	r_1_	r_2_	r_3_	r_4_	Aggregated for r	p	First infection	Peak
**k**	0.230	0.017	0.313	0.216	0.261	0.315	0.077	0.039	0.140	0.537
*π*	0.030	0.03	0.353	0.112	0.545	0.136	0.057	0.793	0.245	0.880
**T_D_**	0.016	0.008	0.138	0.045	0.181	0.047	0.018	0.002	0.062	0.314
$T_{R_{N}}$	0.788	0.030	0.715	0.172	1.034	0.238	0.081	0.128	0.417	1.014
$T_{R_{NN}}$	0.026	0.963	0.646	0.812	0.845	0.747	0.918	0.005	0.724	0.829
$T_{I_{N}}$	0.028	0.052	0.466	0.186	0.700	0.145	0.080	0.004	0.260	0.885

0.5 Values of the total index slightly exceeding 1 are due to MC approximation.
