# Supplementary material for: The first wave of the SARS-CoV-2 epidemic in Tuscany (Italy): A SI2R2D compartmental model with uncertainty evaluation
Source: PLoS One. 2021 Apr 21;16(4):e0250029. doi: 10.1371/journal.pone.0250029 (PMC8059849; doi:10.1371/journal.pone.0250029)
Supplement: S1 Appendix — (PDF) [file pone.0250029.s001.pdf]

# S1 Appendix

## Additional details on parameter definition

The transition parameters $\alpha_{NN}$, $\alpha_N$, $\gamma$ and $\delta$ are daily rates hence they are expressed as ratios of probabilities and times [1, 2]. They can be derived as follows:

$$\alpha_{NN} = \frac{1-\pi^\star}{T_{R_{NN}}} \qquad \alpha_N = \frac{1-h^\star}{T_{R_N}} \qquad \gamma = \frac{\pi^\star}{T_{I_N}} \qquad \delta = \frac{h^\star}{T_D},$$

where $\pi^*$and $h^*$ are the probabilities of being tested and dying for an individual randomly sampled from those who are in the same compartment at the same time. The probabilities $\pi^*$and $h^*$ are related to the probability of being tested for an individual randomly sampled from the total set of the infected ($\pi$) and to the case fatality rate ($h$) as follows [1]:

$$\pi^\star = \frac{\pi T_T}{\pi T_{I_N} + (1-\pi)T_{R_{NN}}} \qquad h^\star = \frac{hT_D}{hT_D + (1-h)T_{R_N}}.$$

## Parametric bootstrap

We perform a parametric bootstrap [3, Ch 6.5] to compute confidence intervals around the point estimates for $\mathbf{r}$ and $h$. Generally speaking, one can rely on the parametric approach when a reasonable estimate of the probability distribution underlying the observed data can be derived from a parametric model. Regarding compartmental models, it is a common practice to assume that the daily increments of deaths and infections are distributed according to Negative Binomial distributions. In particular, we assume:

$$\begin{aligned} i_N(t) &\sim NB(\lambda_i(t), \omega_i(t)) \\ d(t) &\sim NB(\lambda_d(t), \omega_d(t)), \end{aligned} \tag{1}$$

where $NB(\lambda, \omega)$ is a Negative Binomial distribution with mean $\lambda$ and clumping parameter $\omega$.

Starting from the estimates of the model parameters $\widehat{\mathbf{r}}$ and $\widehat{h}$ obtained via calibration, we compute the size of the corresponding SI$^2$R$^2$D compartments as $\widehat{S}, \widehat{I_{NN}}, \widehat{I_N}, \widehat{R_{NN}}, \widehat{R_N}, \widehat{D}$ and, from the estimated time series $\widehat{D}(t)$ and $\widehat{C}(t)$, the daily increments $\widehat{d}(t) = \widehat{D}(t) - \widehat{D}(t-1)$ and $\widehat{i_N}(t) = \widehat{C}(t) - \widehat{C}(t-1)$ $(t = 0, ..., T)$. Then, we set in (1):

$$\begin{aligned} \widehat{\lambda_i}(t) = \widehat{i_N}(t), \ \ \widehat{\omega_i}(t) = \widehat{i_N}(t-1) \\ \widehat{\lambda_d}(t) = \widehat{d}(t), \ \ \widehat{\omega_d}(t) = \widehat{d}(t-1). \end{aligned}$$

It should be noticed that, being the variance of the Negative Binomial equal to $\lambda(1 + \lambda/\omega)$, the distributions (1) account for over-dispersion, especially at the beginning and at the end of the study period, when the number of deaths and new infections is small [5].

In order to get $n = 1000$ bootstrap replications of the model parameters we adopt the following procedure proposed by [4]:

- For each $t$, we sample increments $i^*_N(t)$ and $d^*(t)$ from the following Negative Binomial distributions:

$$\begin{aligned} i^*_N(t) &\sim NB(\widehat{\lambda_i}(t), \widehat{\omega_i}(t)) \\ d^*(t) &\sim NB(\widehat{\lambda_d}(t), \widehat{\omega_d}(t)), \end{aligned} \tag{2}$$

- We derive one bootstrap replication for the cumulative sum of these sampled increments obtaining one bootstrap sample of $D^*(t)$ and $C^*(t)$,
- We perform a calibration procedure assuming the bootstrap time series $D^*(t)$ and $C^*(t)$ as observed, thus obtaining a bootstrap estimate for $\mathbf{r}$ and $h$.

This procedure is repeated $n$ times to obtain $n$ bootstrap replicates of the unknown parameters of the $\mathrm{SI^2R^2D}$ model and compute their percentile intervals. [3, Ch 13]

## Sobol's decomposition of the variance

Let us consider a model which takes $K_X$ mutually independent variables $(X_1, X_2, ..., X_{K_X})$ as input, and returns the output $Y$. According to Sobol's decomposition, the variance of $Y$ can be written as follows [6]:

$$\mathrm{Var}(Y) = \sum_i V_i + \sum_{i<j} V_{ij} + \sum_{i<j<l} V_{ijl} + ... + V_{12...K_X}, \tag{3}$$

where $V_i := \mathrm{Var}(\mathrm{E}(Y|X_i))$ are the first-order variances, $V_{ij} = \mathrm{Var}(\mathrm{E}(Y|X_i, X_j)) - V_i - V_j$ are the second-order variances, and so on. Relying on this variance decomposition, the first-order index for the output $Y$ and the input $X_i$ is defined as the ratio $S_i = \frac{V_i}{\mathrm{Var}(Y)}$. It captures the fraction of the total variance which is attributable to the main effect of $X_i$ on $Y$. The second-order index for the output $Y$ and the inputs $X_i$ and $X_j$ is defined as the ratio $S_{ij} = \frac{V_{ij}}{\mathrm{Var}(Y)}$. It captures the fraction of the total variance which is attributable to the interaction of $X_i$ and $X_j$, and so on. The total effect index introduced in Section 3.4 is defined as:

$$\begin{aligned} S_i^{\mathrm{tot}} &= S_i + \sum_{j>i} S_{ij} + \sum_{h>j>i} S_{ijh} + ... + S_{1,2,...,K_X} = \\ &= \frac{\mathrm{E}(\mathrm{Var}(Y|X_{\sim i}))}{\mathrm{Var}(Y)} = 1 - \frac{\mathrm{Var}(\mathrm{E}(Y|X_{\sim i}))}{\mathrm{Var}(Y)}. \end{aligned} \tag{4}$$

# References


1. Legrand J, Grais RF, Boelle PY, Valleron AJ, Flahault A. Understanding the dynamics of Ebola epidemics. Epidemiology and Infection. 2007;135:610–621.
2. Roosa K, Chowell G. Assessing parameter identifiability in compartmental dynamic models using a computational approach: application to infectious disease transmission models. Theoretical Biology and Medical Modelling. 2019;16:1–15.
3. Efron B, Tibshirani R. An Introduction to the Bootstrap. Boca Raton: CRC press; 1994.
4. Chowell G. Fitting dynamic models to epidemic outbreaks with quantified uncertainty: A primer for parameter uncertainty, identifiability, and forecasts. Infectious Disease Modelling. 2017;2:379–398.

5. Grenfell BT, Bjørnstad ON, Finkenstädt BF. Dynamics of measles epidemics: Scaling noise, determinism, and predictability with the TSIR model. Ecological Monographs. 2002;72:185–202.

6. Sobol IM. Sensitivity estimates for nonlinear mathematical models. Mathematical Modeling and Computational Experiment (Engl Transl). 1993;1:407–414.
